# Cellular and molecular mechanisms of motor neuron death in amyotrophic lateral sclerosis: a perspective

**DOI:** 10.3389/fncel.2014.00241

**Published:** 2014-08-14

**Authors:** Ricardo Tapia

**Affiliations:** División de Neurociencias, Instituto de Fisiología Celular, Universidad Nacional Autónoma de MéxicoMexico City, Mexico

**Keywords:** motor neuron degeneration, Amyotrophic lateral sclerosis (ALS), spinal cord, muscle, skeletal, genetic expression, trophic factors, neuroinflammation

Amyotrophic lateral sclerosis (ALS), which was described since 1869 by Jean Martin Charcot, is a devastating neurodegenerative disease characterized by the selective and progressive loss of upper and lower motor neurons of the cerebral cortex, brainstem and the spinal cord. The result of this loss is a progressive and irreversible paralysis leading to a complete incapacity of movements and finally to respiratory failure, but the cognitive functions are not affected and is not merely the result of aging because may occur at young adult ages. There is still no treatment, prevention or reliable biomarkers of ALS and it is clear that this could only be accomplished, as for other neurodegenerative diseases, through the knowledge of the cellular and molecular pathophysiological mechanisms involved. Research on such mechanisms is, therefore, essential. Although progress in neurochemical, physiological, genetic and clinical investigations in the last decades has identified several cellular processes and mechanisms that seem to be involved in the neuronal death, such as glutamate receptors-mediated excitotoxicity, disruption of spinal inhibitory circuits, inflammatory events, axonal transport deficits, oxidative stress, mitochondrial dysfunction, energy failure, intracellular Ca^2+^ dishomeostasis, protein aggregation and misfolding, changes in gene expression, astrocytes alterations, and non-cell autonomous toxic factors, the understanding of the origin and temporal progress of the disease is still incomplete and insufficient. Clearly, there is a need of further experimental models and approaches to discern the importance of such mechanisms and to discover the factors that determine the selective death of motor neurons characteristic of ALS, in contrast to other neurodegenerative diseases such as Parkinson's and Alzheimer's disease in which other neuronal types located in other CNS regions are predominantly affected.

The only known cause of the disease is associated with genetic mutations, mainly in the gene encoding superoxide dismutase 1 (SOD1) which is the most frequent case of familial ALS (FALS), representing about 20% of cases of FALS, albeit over one hundred mutations have been described in FALS. In contrast, there is no known cause of the sporadic form of ALS (SALS), that comprises >90% of all cases, although several other mutations have recently been identified also in SALS. Both ALS types show similar histopathological and clinical characteristics, and in spite of numerous investigations using tissue from ALS patients and relevant advances in the design of several experimental models of motor neuron degeneration, it has not been possible to establish a clear cause-effect relationship regarding the loss of motor neurons or the motor alterations characteristic of the disease, such as fasciculations, spasticity, and progressive paralysis.

Studies *in vitro* in cell cultures, tissue slices or organotypic preparations have given useful information regarding the cellular and molecular mechanisms of motor neuron death. However, except for transgenic rodents expressing mutant forms of human SOD1 (mSOD1), which are the most extensively used model *in vivo* in spite of the low frequency of FALS as compared to SALS, experiments in living animals are scarce. These models should reflect more closely the situation in the human disease, provided that the symptoms and their development during time mimics as close as possible those of the human disease. It is therefore necessary to correlate the experimental findings *in vitro* with those *in vivo*, as well as those obtained in genetic models with those in non-genetic models, in order to design and test therapeutic strategies based on the results obtained. The aim of the original research articles and reviews of this Research Topic is to contribute to the progress in this important field.

Genetic alterations, including epigenetic and gene expression changes in both neurons and glial cells, mainly astrocytes, as well as studies *ex vivo* of nervous and muscular tissue from ALS patients and from mutant SOD1 transgenic mice, constitute the majority of the articles of this Research Topic. Such changes have been studied in whole tissue, microdissected isolated cells and subcellular fractions, mainly mitochondria, using several molecular biology techniques, not only in the CNS but also in muscle of mSOD mice, and have shown that there is a large variety of alterations in gene expression. These results are the subject of the articles by Heath et al. (2013); Crippa et al. (2013); de Oliveira et al. (2013), and Wong et al. (2013), which amply cover the literature on this subject and also provide new data, such as the relation of some of these genetic alterations with autophagy, epigenetic changes of mitochondrial DNA in the spinal cord and in muscle, and comparative analysis of early and late gene expression changes.

The role of astrocytes and microglia from mSOD mice in non-cell autonomous processes, including the release of toxic factors, is studied by Rojas et al. (2014) and Trias et al. (2013), with a useful *ex vivo* approach. Related studies by Staats et al. (2013) and Bowerman et al. (2013) show the involvement of microglia and neuroimmunological events in the progress of ALS and suggest possible therapeutic strategies. The molecular modifications of mSOD1 protein, and how such alterations, like oxidation, aggregation or misfolding, result in its toxicity, are reviewed by Furukawa (2013); Rotunno and Bosco (2013), and Ogawa and Furukawa (2014). Again in mSOD1 mice but with a different approach, Mòdol et al. (2014) show a possible role of the potassium chloride cotransporter 2 in spasticity, including results on the role of spinal inhibitory interneurons.

A partial protective effect of the NMDA receptor antagonist gacyclidine on the motor deficits in mSOD1 mice is shown by Gerber et al. (2013), and the role of trophic factors in the modulation of motor neurons activity as well as their protective effect in several models of ALS, is reviewed by Tovar-y-Romo et al. (2014) and Lladó et al. (2013). It is emphasized in particular the potent protective action of vascular endothelial growth factor (VEGF). Other potential protective agents, reviewed by Lazo-Gómez et al. (2013), are activators or inhibitors of histone deacetylases, enzymes that regulate gene expression and may be involved in motor neuron degeneration.

A general panorama of the role of lipids in ALS is presented by Schmitt et al. (2014), with emphasis on energy and signaling mechanisms. In a more systemic approach, Garbuzova-Davis and Sanberg (2014) review the vascular changes that may alter blood brain barrier and thus be involved in the pathogenesis of ALS, as compared to the transgenic rodent models.

The large amount of information contained in this Research Topic, in both the comprehensive reviews and the original research articles, reflect the considerable advances made but at the same time the necessity for new experimental models and approaches for gaining clearer responses to several still unresolved questions, such as the following: why the clinical manifestations of motor neuron death in both FALS and SALS appear in young adulthood but are not age-related, particularly the former, in spite of the fact that genetic alterations are present since the beginning of individual life? Why the motor neuron degeneration progresses rapidly after the initial symptoms appear? What is the cause of SALS? It is now clear that the loss of motor neurons in ALS is multifactorial, but many of the deleterious factors seem to occur also in other neurodegenerative diseases like Parkinson's and Alzheimer' disease; then, maybe the crucial question to be answered is: what determines the predominant, if not totally selective characteristic death of motor neurons in ALS, sparing other neuronal types?

In spite of these unsolved questions, it is clear that the available information permits the design and testing of diverse therapeutic strategies that have already shown valuable results in delaying or preventing neuronal death in experimental models, for example the administration of VEGF. However, the translation of possibly effective treatments from experimental procedures to ALS patients represents different problems, such as the routes, doses and timing of administration and the potential undesirable collateral effects. Because we have no indication or biomarker of the susceptibility to ALS and of when will it manifest, it is extremely difficult to prevent or treat the disease. When symptoms have already started and the diagnosis is made it is probably too late to impede the progression of neuronal degeneration. A verifiable resolution of the above questions seems a requisite for efficient and reliable treatments or prevention of this dreadful disease.

## Conflict of interest statement

The author declares that the research was conducted in the absence of any commercial or financial relationships that could be construed as a potential conflict of interest.
